# Reviewed article: Bastos ECL, Vidal NAC, Aragão LS, Costa GMAC, Silveira JAC. Marketing violations involving products that compete with breastfeeding in the vicinity of early childhood education centers and health centers: a cross-sectional study based on an audit ofthe retail food environment, Maceió, 2022-2023. Epidemiol Serv Saude. 2025:34;e20240666

**DOI:** 10.1590/S2237-96222025v34e20240666.b

**Published:** 2025-09-29

**Authors:** Daiane Sousa Melo

**Affiliations:** 1 Universidade de São Paulo, São Paulo, SP, Brazil Universidade de São Paulo São Paulo SP Brazil

## Peer review B

## Questionnaire

Does the manuscript contain new and significant information to justify publication? Yes

Does the Abstract (Summary) clearly and accurately describe the content of the article? Yes

Is the problem significant and concisely stated? Yes

Are the methods described comprehensively? Yes

Are the interpretations and conclusions justified by the results? Yes

Is adequate reference made to other work in the field? Yes

## Manuscript Structure

Length of article is: Adequate

Number of tables is: Adequate

Number of figures is: Adequate

Please state any conflict(s) of interest that you have in relation to the review of this paper. None

Interest: Excellent

Quality: Good

Originality: Excellent

Overall: Good

Recommendation: Minor Revision

## Comments

Parabenizo os autores pelo trabalho desenvolvido com tema relevante para a saúde pública e proteção da amamentação. O manuscrito está escrito de forma clara e objetiva, coloco abaixo algumas sugestões de melhorias pontuais:

Sugiro reduzir o tamanho do título, deixar mais objetivo, por exemplo:

“Infrações comerciais de produtos concorrentes ao aleitamento materno no entorno de centros de educação infantil e unidades de saúde de Maceió/Alagoas"

INTRODUÇÃO

No primeiro parágrafo sugiro exemplificar quais foram os avanços e exemplos dos índices.

No quarto parágrafo sugiro citar o impacto na evolução dos indicadores. Esse poderia ser o primeiro parágrafo do manuscrito para iniciar diretamente abordando a NBCAL.

No objetivo sugiro manter "centros de educação infantil e unidades básicas de saúde" em vez de usar o termo "espaços frequentados por menores de cinco anos". Fazer igual no resumo.

Na Figura 1 sugiro usar uma cor diferente no buffer de 400m para ficar mais fácil visualizar os pontos vermelhos dos comércios com infrações.

Tabela 1. Em: “Presença de qualquer estratégia de promoção comercial "em de" fórmulas infantis, fórmulas infantis de seguimento para lactentes, bicos, chupetas e mamadeiras e protetores de mamilo” excluir “de” que parece estar sobrando.

Sugiro citar exemplo da frase de advertência que os produtos deveriam conter.

RESULTADOS

Sugiro citar nos resultados os nomes das empresas fabricantes.

